# Mass Transport Effects in Suspended Waveguide Biosensors Integrated in Microfluidic Channels

**DOI:** 10.3390/s121114327

**Published:** 2012-10-25

**Authors:** Chaitanya R. Murthy, Andrea M. Armani

**Affiliations:** Mork Family Department of Chemical Engineering and Materials Science, University of Southern California, 3651 Watt Way, Los Angeles, CA 90089, USA; E-Mail: cmurthy@usc.edu

**Keywords:** integrated biosensor, finite element simulations, sample delivery, microfluidics

## Abstract

Label-free optical biosensors based on integrated photonic devices have demonstrated sensitive and selective detection of biological analytes. Integrating these sensor platforms into microfluidic devices reduces the required sample volume and enables rapid delivery of sample to the sensor surface, thereby improving response times. Conventionally, these devices are embedded in or adjacent to the substrate; therefore, the effective sensing area lies within the slow-flow region at the floor of the channel, reducing the efficiency of sample delivery. Recently, a suspended waveguide sensor was developed in which the device is elevated off of the substrate and the sensing region does not rest on the substrate. This geometry places the sensing region in the middle of the parabolic velocity profile, reduces the distance that a particle must travel by diffusion to be detected, and allows binding to both surfaces of the sensor. We use a finite element model to simulate advection, diffusion, and specific binding of interleukin 6, a signaling protein, to this waveguide-based biosensor at a range of elevations within a microfluidic channel. We compare the transient performance of these suspended waveguide sensors with that of traditional planar devices, studying both the detection threshold response time and the time to reach equilibrium. We also develop a theoretical framework for predicting the behavior of these suspended sensors. These simulation and theoretical results provide a roadmap for improving sensor performance and minimizing the amount of sample required to make measurements.

## 1. Introduction

Label-free optical biosensors based on integrated photonic devices are able to accurately detect chemical and biological molecules over a wide concentration range in real-time [1–4]. Therefore, these devices have a broad range of applications, spanning from defense to the pharmaceutical industries. For example, they have been used to determine the kinetic constants (binding coefficients) of many receptor:ligand pairs [5] and in the development of sensors for monitoring environmental biohazards [1,6].

The performance of an integrated label-free biosensor is typically characterized according to its sensitivity or limit of detection for a given analyte of interest, with researchers continuously pushing the boundaries on sensitivity. In parallel, moderate consideration is usually given for specificity through the development of surface chemistries for targeting the analyte of interest. However, minimal effort is expended on optimizing the collection efficiency of the sensor. By increasing the collection efficiency, it is possible to significantly reduce the amount of sample required for a measurement and compensate for a lower sensitivity device. Minimizing sample consumption is critical for analytical applications, particularly those involving rare or valuable materials. As such, any improvements to the collection efficiency can reduce the cost and increase the ease of conducting experiments that allow for further optimization of sensitivity and specificity. Therefore, because of the balance between sensitivity and collection efficiency, the optimization of collection efficiency and the device sensitivity should occur in parallel.

**Figure 1.**
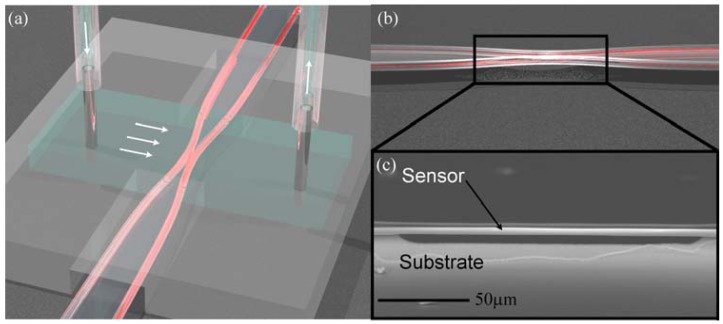
Suspended waveguide splitter/coupler biosensor. (**a**) Rendering of the biosensor integrated into a simple microfluidic flow cell. Light confined within the waveguide is shown in red, and the flow cell is shaded blue-green. The arrows indicate the direction of the fluid flow, which is perpendicular to the waveguide, interacting only with the suspended splitter/coupler region; (**b**) Rendering of the waveguide splitter/coupler device highlighting the energy transfer between the two coupling/splitting arms of the device; (**c**) Side view scanning electron micrograph (SEM) of the suspended region of the waveguide splitter/coupler.

In the present work, we explore how the vertical placement of a suspended optical device within a microfluidic channel can influence its collection efficiency through a series of finite element method simulations. This work is focused on integrated waveguide biosensors, which have demonstrated the ability to detect bacteria, cells and proteins in complex environments [7–10]. As a result of the fabrication design, all of these waveguides were located directly on the substrate. Recently, a new type of waveguide sensor was developed that is elevated off of the substrate (Figure 1), offering the possibility of improved sample delivery [11,12]. Previous research on electrical nanowire sensors has demonstrated improved collection efficiency using this approach [13]. By considering fluid flow and monitoring the surface concentration of bound analyte over a range of system parameters, we are able to compare the sample delivery efficiency of this new suspended sensor to that of more established waveguide sensor geometries. We also develop a general theoretical framework for analyzing the response characteristics of suspended waveguide biosensors.

## 2. Details of the Model

In order to accurately determine the collection efficiency of the suspended waveguide device, it is necessary to account for both the fluid flow around the sensor and the reaction kinetics at the surface of the sensor. This type of complex, interdependent modeling is ideally suited for COMSOL Multiphysics, a finite element simulation package, which can incorporate multiple physical phenomena interactively.

**Figure 2.**
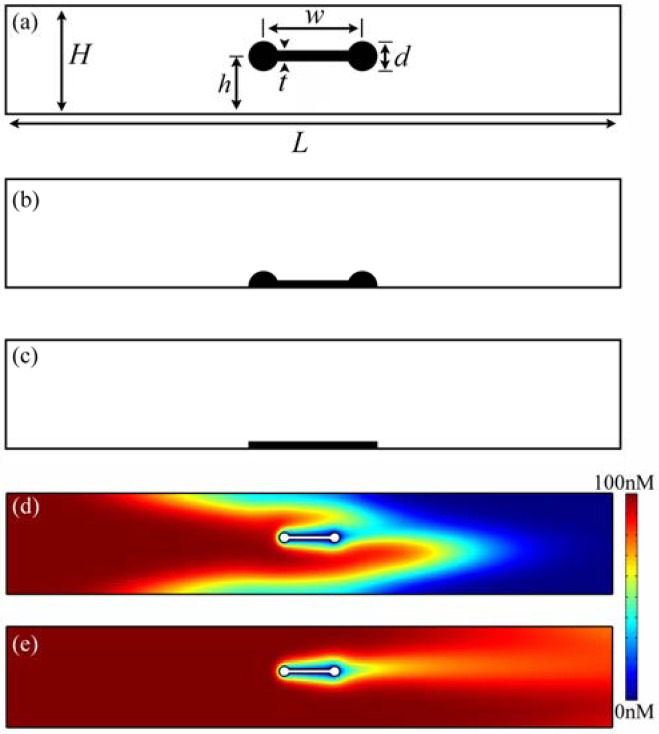
(**a**) Schematic of a 2-D cross-section of the flow cell and suspended optical waveguide sensor (not to scale). The sensor is located in the middle of the flow cell lengthwise. The flow cell height *H* = 50 μm, length *L* = 300 μm; waveguide width *w* = 25 μm, diameter *d* = 5 μm, membrane thickness *t* = 2 μm, and elevation *h* = 25 μm. Different values of *H* and/or *h* were also used for some studies (where indicated). (**b**) Waveguide sensor embedded at its mid-plane in channel floor (corresponding to *h* = 0 μm). All other geometry parameters are identical to those in (**a**), and the sensor protrudes *d*/2 = 2.5 μm into the flow stream. (**c**) Flat planar sensor located on channel floor. The sensor has the same footprint as the other geometries, but does not protrude into the channel at all. (**d**) & (**e**) Transient concentration profiles showing analyte flowing into the channel and the development of a concentration depletion region around the suspended sensor. The images are from a COMSOL simulation with parameters: *h* = 28 μm, *v̅_in_* = 2 × 10^−4^ m/s, [A]_0_ = 100 nM, [B]*_m_* = 1.66 × 10^−9^ mol/m^2^, and others as in Table 1, at times 0.8604 s and 2.76 s respectively. The concentration scale bar is the same for both FEM images and ranges from 0 (dark blue) to 100 nM (red). Fluid flows through the cell from left to right in all panels.

Specifically, finite element method simulations were performed using COMSOL Multiphysics 4.2 to solve the Navier-Stokes (*i.e.*, momentum balance and continuity equations) and conservation of mass equations in a geometry representing a microfluidic flow cell containing a suspended optical waveguide sensor oriented transverse to the flow (Figure 2(a)). All dimensions were based on experimentally realistic conditions or previously determined values. For example, to accurately determine the dimensions of the optical waveguide, we used the results from previous work which first demonstrated this suspended waveguide device [12]. Similarly, the total height *H* of the channel was varied between 25 and 100 μm to reflect typical polydimethylsiloxane (PDMS) microfluidic device dimensions [14,15].

In order to compare sample delivery efficiencies of substrate-bound and suspended sensors, we varied the vertical position *h* of the suspended sensor within the channel (Figure 2(a)). This height is determined by the combination of isotropic and anisotropic etchants which are used in the device fabrication process; therefore, there is extremely good control (sub-μm) over this parameter. The precise range of attainable values of *h* has yet to be determined for this young technology, but values in excess of 50 μm are routinely achieved. The elevation values we consider here fall within the experimentally demonstrated range of values of *h*, and also include the limiting value of zero elevation (Figure 2(b)). We also modeled a simple rectangular sensor that is embedded in the channel floor (Figure 2(c)). This geometry is similar to that of slab waveguide sensors and surface plasmon resonance (SPR) sensors. The flat SPR sensor, first commercialized by Biacore, is a benchmark for comparison, and also serves to verify our results with past studies [16–19].

A finite element mesh was generated to focus computation power on regions of the flow cell where the dependent variables were most influenced by position. The model was tested over a range of mesh element sizes to check for convergence and to ensure that the model had sufficient spatial resolution to capture relevant phenomena. The accuracy of our model was determined based on its ability to reproduce analytical results for simple cases. Additional details of how the computational model was built and validated, along with specifics of convergence tests and details of the finite element mesh used are included in the online supplementary information.

Several assumptions were made in order to simplify the process of solving for the fluid velocity and analyte concentration profiles in the system. First, the 3-D geometry was reduced to the 2-D cross section along the length of the channel shown in Figure 2. This is an acceptable approximation when the effective sensing area of the waveguide is situated in the middle of the channel and away from the sidewalls, as it is for the devices of interest, or when the channel width is very large relative to the channel height [20]. The simulated channel extends six sensor widths upstream and six widths downstream from the sensor in the direction of fluid flow, for a total length of 300 μm. Additionally, we consider only binding of the analyte to the functionalized sensor and ignore any non-specific adsorption to the sensor or channel walls. This simplification is supported by recent advances in surface functionalization chemistry for gold and silica surfaces that significantly reduce the amount of non-specific binding [21–23]. We further assume incompressible, laminar flow that enters the channel with a fully developed parabolic velocity profile. Since this inlet flow profile is symmetric about the mid-height of the channel, it is only necessary to consider sensor elevations ranging from mid-channel to the flow cell floor.

It is important to note that the parabolic flow profile is characteristic of a pressure-driven flow, which is the conventional method used for PDMS microfluidic channels [24–26]. An alternative method is electrokinetic-driven flow. The flow profile for electrokinetic flow is inverted, with the high flow velocity on the boundaries and slower flow in the middle of the channel [27]. However, electrokinetic flow requires a fluid that contains charged molecules. As a result, when comparing the maximum achievable flow rates and utility of the two methods, it is widely acknowledged that pressure driven flow is able to achieve higher flow rates and is applicable to a broader range of biological fluids [28,29]. Therefore, we have focused our efforts on pressure-driven flow.

The adsorption of analyte to the sensor surface is approximated as (first-order) Langmuir binding [30] according to the following reaction between a freely diffusing protein A with concentration [A] and an unoccupied binding site B with surface concentration [B] forming a bound complex *C_s_* with surface concentration [C_s_]:

(1) $$A+B↔C_{s}$$

Mass action kinetics allow us to express the rates of the forward and reverse reactions in terms of the forward and reverse kinetic rate constants *k_f_* and *k_r_*, respectively, as:

(2) $$\text{rat}e_{f}=k_{f}[A][B]$$

(3) $$\text{rat}e_{r}=k_{r}[C_{s}]$$

where the *dissociation* equilibrium constant is *K_D_* = *k_r_/k_f_*. Each bound analyte molecule is assumed to occupy a single binding site. Equations (2) and (3) can be used to relate the concentration of freely diffusing analyte at the sensor surface to the surface concentration of the bound species, a boundary condition based on the conservation of mass (see Equation (S13) in the online Supplementary Information).

Interleukin 6 and anti-IL6.8 were chosen as the representative analyte and receptor, respectively. For this system, we used the parameters listed in Table 1, varying certain parameters for different studies. Interleukin 6 was chosen because it is involved with a range of important biologic functions. For example, it plays a key role in the immune and neural systems, in hematopoiesis, and in acute phase response, where it is a sensitive physiological marker of systemic inflammation [31,32]. Representative concentration profiles are shown in Figure 2(d,e).

It is important to note that in optical devices, additional phenomena are present which can enhance the collection of particles by the sensor surface, including photophoresis and thermophoresis [33–35]. The presence of these forces has been observed with micron-sized nanoparticles. However, the present work is studying IL-6, which is a nano-scale biological particle with minimal charge. Therefore, based on the fundamental governing equations for the forces, the effect would be negligible [36–38].

We investigated two critical measures of sensor response time to characterize the collection efficiency: (1) equilibration time and (2) time of detection. For the present work, the equilibration time *t_eq_* was defined as the length of time required for the surface concentration of bound complex to reach 95% of its equilibrium value. This arbitrary but convenient percentage was chosen to minimize the error in identifying this threshold time that can arise as the surface concentration asymptotically approaches its equilibrium value, while still giving a realistic approximation of how long the device takes to equilibrate. The limit of detection [C_s_]_min_, and corresponding detection time *t_d_*, represent the lowest concentration of surface-bound analyte that can be detected, and the length of time taken to achieve this value, respectively. In our studies, [C_s_]_min_ was set at 10 pg/mm^2^ (equivalently 3.85 × 10^−10^ mol/m^2^, based on a representative molecular weight of 26 kDa for IL-6), which corresponds to approximately 2.5% coverage of the suspended sensor surface. This conservative value was selected based on the current experimental results using waveguide biosensors [7–10]. Both of these metrics are commonly used in characterizing the performance of biosensors and other types of chemical detectors [2].

In characterizing the sensor response, we also accounted for the time delay that may occur as analyte is carried from the flow cell inlet to the sensor via advection. For example, at *v̅_in_* = 10^−4^ m/s, it would take ∼1 s for the antigen molecules introduced at the flow-cell entrance at time zero to travel 150 μm downstream to the waveguide and begin binding. We defined the start of binding as the time *t*_1_ when the average surface concentration is equivalent to a single molecule of bound analyte per micrometer of sensor length (for details, see Section 3.1 of the supplementary information).

**Figure 3.**
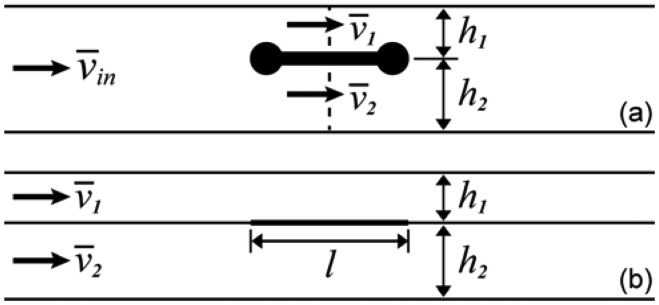
(**a**) Schematic of the suspended optical waveguide sensor in the flow cell (not to scale). *h_1_* and *h_2_* are defined as the distances from the mid-plane of the sensor to the top and bottom surfaces of the channel, *v̅*_1_ and *v̅*_2_ are the average flow velocities through the regions above and below the sensor, and *l* is the length of the sensor in the direction of flow; (**b**) Schematic of an analogous hypothetical system consisting of two identical surface-bound flat planar sensors of length *l*, located in two channels of height *h_1_* and *h_2_* respectively, through which fluid flows with average velocity *v̅*_1_ and *v̅*_2_ respectively. Our approach is built on the intuition that the binding behavior of the suspended sensor might be reasonably approximated by the sum of the binding behaviors of these two flat planar sensors, which represent its upper and lower surfaces.

The problem of convection, diffusion and reaction to traditional surface-bound flat planar sensors has been studied extensively, both via simulation and experiment [44,45], and powerful theoretical methods exist to characterize the regimes of operation of such devices based on the values of a few key dimensionless numbers, and thereby approximate their binding behavior over a vast range of operational parameters [20]. Our general approach to analyzing suspended biosensor behavior in microfluidic channels builds upon this knowledge and intuition by treating the upper and lower surfaces of the suspended sensor as individual flat planar sensors located in an analogous two-channel system (Figure 3(a,b)). This analytical approach complements our simulation results in providing insights into the probable behavior of the new sensor geometry.

## 3. Results and Discussion

### 3.1. Velocity and Height Dependent Fluid Flow Simulations

We begin by discussing flow around the suspended sensor. Consider the average velocity of fluid in the regions directly above and below the sensor (*v̅*_1_ and *v̅*_2_ respectively). In general, *v̅*_1_ and *v̅*_2_ depend on the size of the gaps *h_1_* and *h_2_*, on the geometry of the sensor, and on the Reynolds number of the incoming flow:

(4) ReH=ν¯inHν

where *ν* = *μ*/*ρ* is the kinematic viscosity (dynamic viscosity divided by density) of the fluid, and the subscript *H* specifies the total channel height as the relevant length scale. When Re « 1 and *l*/*H* is large, the pressure drop per unit length along each hypothetical channel in Figure 3(b) is identical and the average velocity in a channel varies as the square of the height. Together with a mass balance over the entire system, this gives in the “slow limit”:

(5) $$\frac{{\bar{\nu }}_{1}}{\bar{\nu }}=\frac{{\left(h_{1}/H\right)}^{2}}{{\left(\overset{h_{1}}{\;}/\underset{H}{\;}\right)}^{3}+{\left(1-\overset{h_{1}}{\;}/\underset{H}{\;}\right)}^{3}}$$

where *v̅* is the total average velocity of fluid through the dotted line in Figure 3(a). Conversely, when Re » 1 and *l*/*H* is small, streamlines near the sensor do not deviate significantly from their original direction as they pass it, and the rate of fluid flow above or below the sensor can be determined simply by integrating the relevant portion of the incoming parabolic velocity profile. Thus, in the “fast limit”:

(6) $$\frac{{\bar{\nu }}_{1}}{\bar{\nu }}=3\left(\frac{h_{1}}{H}\right)-2{\left(\frac{h_{1}}{H}\right)}^{2}$$

**Figure 4.**
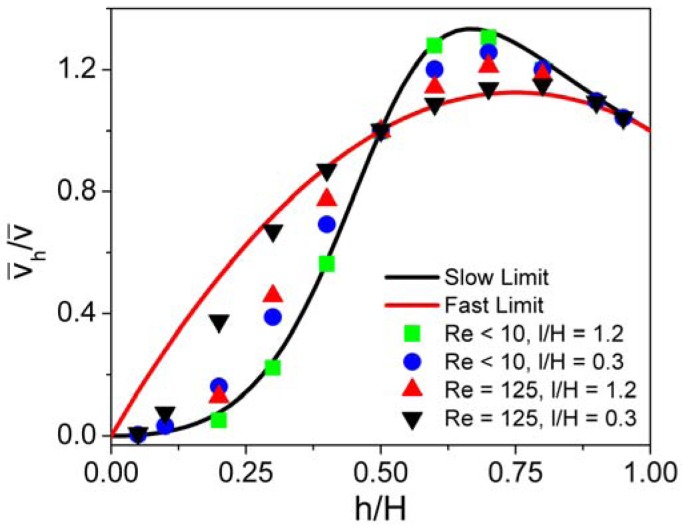
Average flow velocity in the region above or below the sensor as a function of the size of the gap on that side relative to the total channel height. The slow and fast limit expressions are compared with simulation data for a range of Reynolds numbers and geometry parameters.

For a derivation of Equations (5) and (6), see the Supplementary Information, Section 4. Figure 4 compares the results obtained in our FEM simulations with those estimated using Equations (5) and (6). In general, the equations provide better relative estimates of the flow velocity as *h*/*H* increases. For Re*_H_* below ∼10 and *l*/*H* > 0.5, the flow velocity on either side of the sensor is approximated well by Equation (5). Conversely, for Re*_H_* greater than ∼100 and *l*/*H* < 1, Equation (6) is reasonably accurate, except at very small values of *h*/*H*. As expected, the slow limit approximation improves with increasing *l*/*H*, and the fast limit approximation with decreasing *l*/*H*.

### 3.2. Incorporation of Mass Transport and Surface Reaction into Fluid Flow Simulations

Next we consider mass transport. The dimensionless flux (Sherwood number) is a useful generalized metric of the rate at which mass transport can deliver analyte molecules to the sensor surface [46]:

(7) $$F=\frac{J_{D}}{D{\left[A\right]}_{o}}$$

where *J_D_* is the total diffusive flux of analyte per unit length to the sensor surface. The nature of mass transport to the sensor is determined by the relative strengths of convection and diffusion in the fluid. A family of Péclet numbers exists that quantify this ratio; they differ from one another based on the relevant length scales over which each effect is considered. We define a “collection” Péclet number:

(8) $$Pe_{c}=\frac{\text{diffusive time}}{\text{convective time}}=\frac{h^{2}{\bar{\nu }}_{h}}{lD}$$

This number compares the time needed for a particle to diffuse across a channel of height *h* with the time needed to convect past a sensor of length *l* at average flow velocity, *v̅_h_*. When Pe*_c_* < 1, convection is slow enough that all analyte molecules flowing past the sensor have enough time to diffuse to the surface. On the other hand, when Pe*_c_* » 1, only analyte molecules in a narrow region near the sensor surface have a chance of being collected as they are swept by.

In contrast to the embedded sensor, whose operation is characterized by a single Pe_c_ value, separate Pe*_c_* values characterize mass transport to the top and bottom surfaces of the suspended sensor. Thus, three distinct mass transport regimes are possible. When Pe*_c_* » 1 on both sides of the sensor, we observe the development of a concentration boundary layer (visible in Figure 2(e)), where the concentration gradient established at the sensor surface due to specific binding exists only over a region of thickness δ ∼ *l* Pe_s_^−1/3^. Here:

(9) $$Pe_{s}=\frac{6{\bar{\nu }}_{h}l^{2}}{Dh}$$

is a second Péclet number that depends on the shear rate past the sensor. The (dimensionless) flux through this depletion region is given by [20]:

(10) $$F\approx {{Pe}_{s}}^{1/3}$$

Conversely, when Pe*_c_* < 1 on both sides of the sensor, all analyte molecules are collected, and the flux on either side is simply:

(11) $$F\approx \frac{{\bar{\nu }}_{h}h}{D}\equiv Pe_{h}$$

Finally, we have the more complex scenario where Pe*_c,1_* < 1 and Pe*_c,2_* » 1. Here, a concentration boundary layer forms below the sensor, and the flux to this side is given by Equation (10). Meanwhile, all target molecules that enter the region above the sensor are collected. However, this scenario requires that the flow rate above the sensor be orders of magnitude smaller than the flow rate below it. Thus, the bulk flow deviates around the gap and target molecules are primarily pulled into the region above the sensor by diffusion. Consequently, the flux to the upper surface of the sensor is given by

(12) $$F\approx {\left(\frac{6{\bar{\nu }}_{in}h_{1}^{2}}{DH}\right)}^{1/3}\equiv {{Pe}_{g}}^{1/3}$$

where Pe*_g_*, a close relative of Pe*_s_*, depends on the shear rate past the gap above the sensor. *h*_1_ is the height of the upper channel (equivalently the height of the gap) and *v̅_in_* is the average fluid velocity of the bulk flow.

For a given set of model parameters, we can easily calculate the equilibrium concentration, [C_s_]*_eq_*, by setting Equations (2) and (3) equal to one another and noting that [B] = [B]*_m_* – [C_s_]:

(13) $${\left[C_{s}\right]}_{eq}=\frac{{\left[A\right]}_{o}{\left[B\right]}_{m}}{K_{D}+{\left[A\right]}_{o}}\equiv {\left[B\right]}_{m}\frac{{\left[A\right]}_{o}/K_{D}}{1+{\left[A\right]}_{o}/K_{D}}$$

Given enough time and an adequate supply of analyte, the concentration of analyte bound to the sensor surface will invariably approach this value. A quantitative analysis of convergence to [C_s_]*_eq_* in our model is included in the online supplementary information. The dissociation equilibrium constant *K_D_* = 6.67 × 10^−12^ M arises as a natural concentration scale. When [A]_0_/*K_D_* » 1, the analyte solution is concentrated enough to essentially saturate the sensor, and [C_s_]*_eq_* ≈ [B]*_m_*. Conversely, when [A]_0_/*K_D_* « 1, only a fraction [C_s_]*_eq_*/[B]*_m_* ≈ [A]_0_/*K_D_* of the available binding sites are bound in equilibrium. Thus, our detection limit estimate of 2.5% sensor surface coverage restricts us to inlet analyte concentrations [A]_0_ > 0.025 *K_D_* or [A]_0_ > ∼200 fM. However, since this estimate is highly conservative, it is possible that we will be able to detect solutions several orders of magnitude more dilute.

The approach to equilibrium is characterized by the Damköhler number, which is defined as the ratio of reactive flux to diffusive flux at the sensor surface [20]:

(14) $$Da=\frac{k_{f}{\left[B\right]}_{m}l}{DF}$$

Here *D* is the bulk diffusion coefficient of IL-6 in water. When Da « 1, the reaction itself is rate-limiting and the concentration of surface-bound analyte grows according to:

(15) $$\frac{\left[C_{s}\right]}{{\left[C_{s}\right]}_{eq}}={1-e}^{-t/\tau_{R}}$$

where:

(16) $$\tau_{R}=k_{r}^{-1}{\left(1+{\left[A\right]}_{o}/K_{D}\right)}^{-1}$$

is the reaction time-scale. At high concentrations ([A]_0_/*K_D_* » 1), Equation (16) reduces to τ*_R_* ≈ (*k_f_* [A]_0_)^−1^ and the reaction time-scale merely reflects how quickly the forward reaction proceeds. On the other hand, at low concentrations ([A]_0_/*K_D_* « 1), the reaction time-scale is determined entirely by the rate of the reverse reaction (τ*_R_* ≈ *k_r_*^−1^). When Da » 1, kinetics are mass transport-limited and the surface concentration increases linearly with time as analyte is delivered to the sensor surface. The binding time-scale in the transport-limited case is Daτ*_R_*.

**Figure 5.**
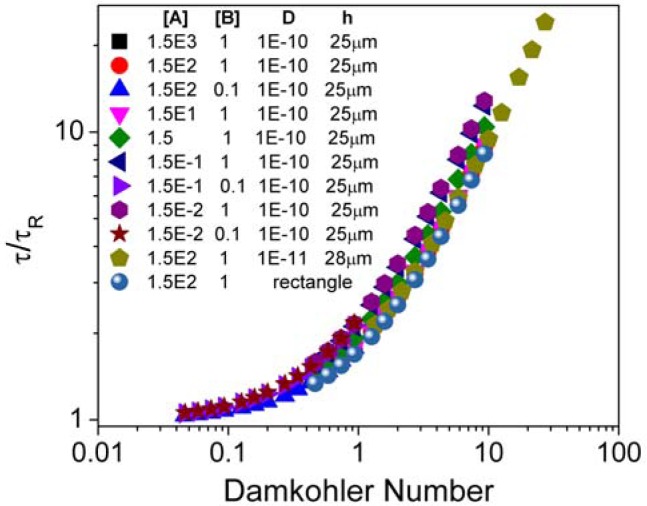
Binding time *τ*, normalized with respect to the reaction-time scale τ*_R_*, plotted as a function of the Damköhler number Da. By analog with the definition of *τ_R_* in Equation (16), we define *τ* as the time taken to reach 1 − e^−1^ ≈ 63% of the equilibrium concentration. Results from a representative subset of the studies performed are presented, where the channel height *H* = 50 μm. As expected, the ratio τ/*τ_R_* approaches 1 as Da goes to zero, and is approximately equal to Da in the opposite limit. A rectangular suspended sensor was also modeled to demonstrate that these results are independent of the precise sensor geometry.

To analyze the binding response of the present sensor, we performed simulations over a range of conditions with the sensor located at the mid-plane of the channel. Representative results are plotted in Figure 5. As is the case for flat planar sensors, a wide range of analyte concentrations, antibody concentrations, and flow velocities collapse onto a single master curve. These results hold as long as the sensor is located near enough the channel mid-height that the top and bottom surfaces equilibrate on similar timescales. To a reasonable approximation, this is the case when the collection Péclet numbers on either side of the sensor are of the same order of magnitude. Equation (8), together with the slow-limit result that the average fluid velocity varies as the square of the channel height, gives us that Pe*_c_* ∼ *h^4^*. Therefore, the binding response of any sensor suspended in a channel such that *h_1_* and *h_2_* differ by a factor of ∼1.8 or less (or equivalently, 0.35 < *h*/*H* < 0.65) should be well approximated by the mid-channel results.

The preceding analysis and the master curve of Figure 5 apply throughout most of the parametric domain of Table 1 for sensors near mid-channel. Our simulation results only showed deviations in those regimes where the assumptions underlying our quasi-steady analysis break down; where the time needed for the boundary layer to develop is sufficient to appreciably saturate the sensor. This occurs at the highest inlet analyte concentrations, low flow velocities and low surface antibody concentrations.

### 3.3. Effect of Sensor Height and Comparison of Sensor Geometries

**Figure 6.**
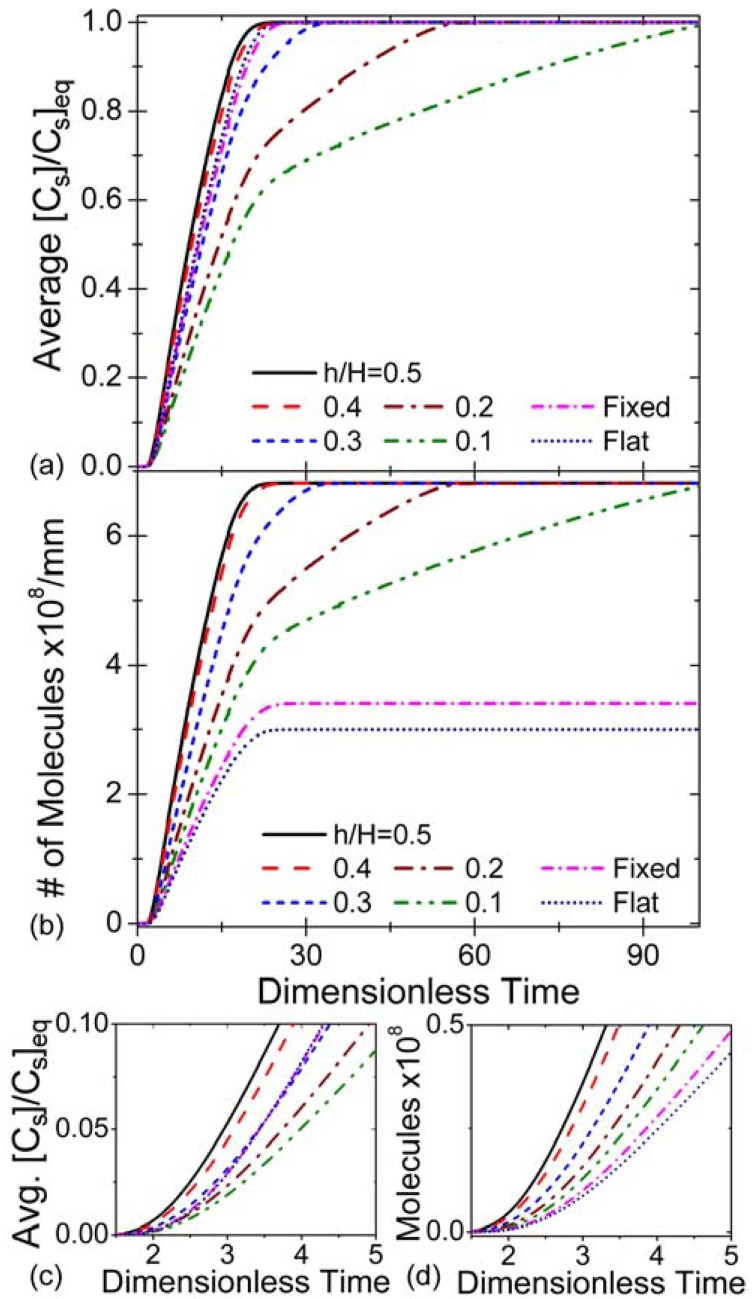
Binding curves for various sensor geometries (inlet bulk analyte concentration [A]_0_ = 100 nM, total surface concentration of binding sites [B]*_m_* = 1.66 × 10^−8^ mol/m^2^, and average inlet flow velocity *v̅_in_* = 5 × 10^−5^ m/s). All times are normalized with respect to τ*_R_*. Consult Figure 2 for schematics of the geometries compared. (**a**) Binding sites on each sensor that are occupied as a function of time, as a fraction of the number of binding sites that would be occupied at equilibrium. (**b**) Number of analyte molecules bound per millimeter length of each sensor geometry as a function of time. (**c**) & (**d**) Subset of the data in graphs (**a**) and (**b**) respectively, highlighting the initial binding behavior of the sensors.

Figure 6 shows binding behavior for various sensor elevations and geometries at a fixed flow velocity. As can be observed, the mid-channel placement reduces binding time amongst the suspended sensors. Additionally, due to the greater than 2-fold increase in sensing surface area, the total mass of IL-6 that can be collected and detected is significantly increased in the suspended geometry, enabling improved collection efficiency and performance (Figure 6(b)). At low flow velocities, advective mass transport to regions beneath the low-elevation sensors becomes insignificant compared to diffusive transport. This is due to the preferential flow of fluid through the much larger gap on the other side of the sensor (as predicted by the analysis in Section 3.2), and leads to the characteristic two-stage binding curves of Figure 6, which feature a regime where advective transport quickly leads to saturation of the antibodies on the top of the sensor surface and another where diffusive transport slowly delivers antigen to the bottom of the device. The upper surfaces all approach equilibrium at approximately the same time (t/τ*_R_* = 20, in this case), but the lower surfaces equilibrate on progressively slower timescales as elevation is reduced. This indicates that suspended sensors with low elevations would be particularly inefficient for making endpoint measurements.

**Figure 7.**
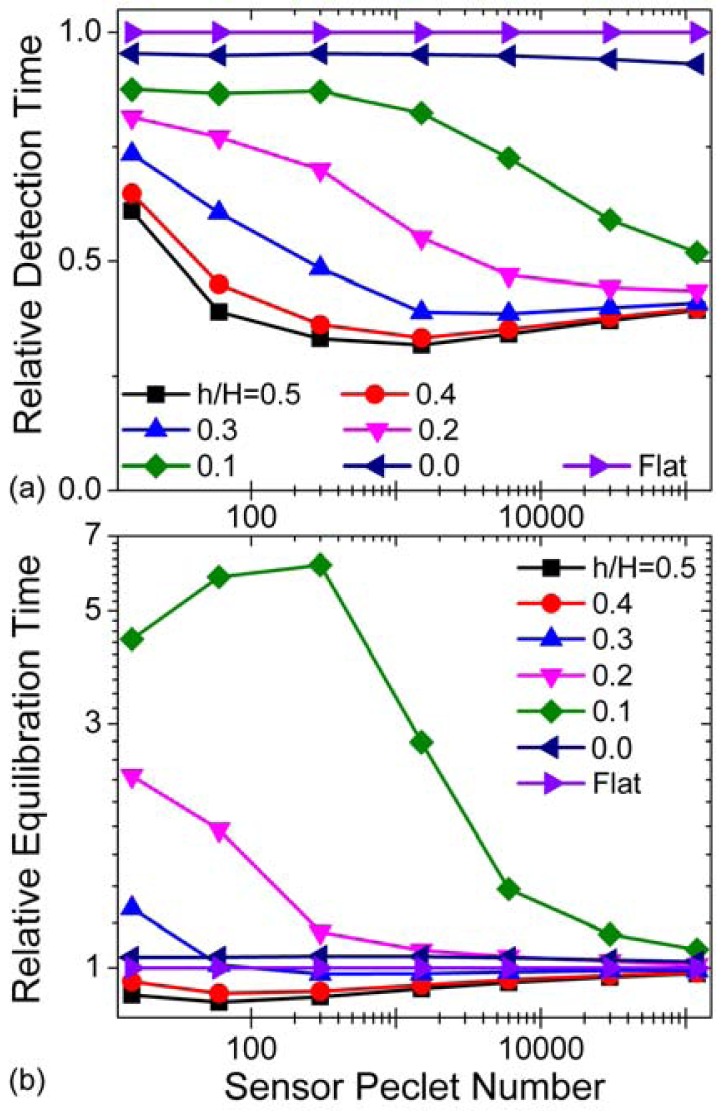
Response times as a function of sensor elevation/geometry and Sensor Péclet Number Pe*_l_* = *v̅_in_l*/*D* (inlet bulk analyte concentration [A]_0_ = 100 nM and total surface concentration of binding sites [B]*_m_* = 1.66 × 10^−8^ mol/m^2^). Note that in these simulations, *l* and *D* were not varied, and the range of average inlet velocities considered was chosen based on probable operating conditions for the waveguide sensor. Cf. Figure 2 for the schematics of the geometries compared. (**a**) Relative detection time: ${\tilde{t}}_{d}^{*}=(t_{d}-t_{1,\text{min}})/(t_{d,\text{flat}}-t_{1,\text{min}})$; **(b)** Relative equilibration time: ${\tilde{t}}_{eq}^{*}=(t_{eq}-t_{1,\text{min}})/(t_{eq,\text{flat}}-t_{1,\text{min}})$.

Figure 7 compares response times of these sensors over a range of flow velocities. To accurately compare the different geometries, we first adjust all response times at a given flow velocity by the smallest *t_1_* for any of the sensors considered. This ensures that we maintain an even reference frame for direct comparisons between the different elevations/geometries. Then, we divide all times by the corresponding flat sensor response time, since this geometry serves as our benchmark. Because the suspended sensor is still in the early stages of its development, the noise-limited detection threshold for this device is unknown. In order to facilitate our discussion, we assume a detection threshold of 0.68 pg/mm, which corresponds to 1.6 × 10^7^ bound antigen molecules per millimeter length of sensor, irrespective of geometry. This particular value is equivalent to our earlier specification of [C_s_]*_min_* for the suspended sensors, but the general trends shown in Figure 7(a) persist over a range of detection threshold values (see Section 3.2 in the online Supplementary Information).

The suspended sensor significantly outperforms the substrate-bound sensor in terms of detection time. At all but the slowest modeled flow velocities, the mid-channel placement offers a greater than twofold reduction in relative detection time over the conventional planar geometry. A long flow cell upstream of the sensor will diminish this performance difference by increasing all *absolute* detection times concurrently. Quicker response times also imply lower sample consumptions for the suspended sensor, especially when paired with a short flow cell. Figure 7(b), meanwhile, shows that suspended waveguide sensors located near the middle of the flow cell and conventional flat planar sensors with equal surface antibody concentrations equilibrate on very similar timescales. However, over twice as many antigen molecules are bound per unit length to the suspended sensors than the flat one at equilibrium, and thus the suspended sensor is able to utilize a larger fraction of the total antigen molecules in the flow cell and should generate a correspondingly larger signal. In addition, the suspended waveguide device should see further performance increases because a larger proportion of the bound antigen molecules interact with the evanescent field of the confined light [11].

## 4. Conclusions

We have developed a finite element model that simulates advection, diffusion and specific binding of IL-6 to the antibody-functionalized surface of a novel suspended waveguide biosensor in a microfluidic channel. We use this model to characterize sensor behavior for a range of average flow velocities, inlet antigen concentrations, and surface-immobilized antibody concentrations. Device performance is evaluated according to two common sensor metrics: the detection time and the equilibration time.

Our model predicts that the detection and equilibration times will have a weak dependence on flow velocity, while inlet analyte concentration can greatly impact the kinetic response of the device. Our results further show that reducing the surface antibody concentration can extend the range of conditions over which the sensor is reaction-limited. However, transport-limited conditions may be reached at sufficiently low flow rates, high analyte concentrations and high antibody concentrations on the surface. These conditions can lead to long equilibration times, require significant sample volumes in order to make measurements, and make it difficult to determine kinetic rate constants for the surface binding reaction, making them ill-suited for most biosensor measurements.

We also compare the specific binding to waveguide sensors suspended at varying heights in the channel with that for planar sensors on the channel floor. The sensor suspended at mid-height in the channel shows shorter detection times than the flat device due to its thinner boundary layers and increased active sensing area. Though equilibration times are similar for these two geometries, sensors elevated only slightly above the channel floor yield significantly slower equilibration times than either the planar substrate or the sensor at mid-height of the channel. The small gap between the suspended sensor and the channel floor at small elevations hinders the efficient mass transport via advection to the bottom side of the device. Our results indicate that suspended sensors can display a range of transient behaviors, with efficiencies either greater than or less than those of traditional planar sensors, depending upon their elevation. Given that lithographic methods are used to fabricate these and other semi-conductor based optical sensors, the elevation of the sensor within the channel can be optimized to locate the device within the region of highest sample delivery to ensure the largest possible performance improvements.

These findings, together with the general framework developed in this paper for analyzing and predicting suspended sensor behavior, provide a basis for optimizing the performance of novel suspended waveguide biosensors in microfluidic channels, leading to reduced sample consumption and improved response time of these devices.

## Supplementary Material



## Figures and Tables

**Table 1.** Model parameters.

**Parameter**	**Description**	**Value/Range**
*k_f_*	Association rate constant	9 × 10^6^ L/mol·s a
*k_r_*	Dissociation rate constant	6 × 10^−5^ s^−1^^a^
*K_D_*	Dissociation equilibrium constant	6.67 × 10^−12^ M
[B]*_m_*	*Total* surface concentration of binding sites on the sensor (also referred to as antibody concentration)	1.66 × 10^−9^ mol/m^2^ to 1.66 × 10^−8^ mol/m^2^^a^
*D*	Bulk diffusion coefficient of IL-6 in water	1 × 10^−10^ m^2^/s a
[A]_0_	Inlet concentration of IL-6 in the bulk solution	10^−13^ M to 10^−7^ M
*v̅_in_*	Inlet average fluid velocity	5 × 10^−5^ m/s to 0.4 m/s

a Rate constant values are from Rispens *et al.*[39].

b Upper limit of the range was estimated from the measured size of a single human IgG antibody [40].

c Estimated from data on diffusion coefficients as a function of molecular weight [41,42] and IL-6 protein mass data [43].
