# Supplementary material for: Mass Transport Effects in Suspended Waveguide Biosensors Integrated in Microfluidic Channels
Source: Sensors (Basel). 2012 Oct 25;12(11):14327–43. doi: 10.3390/s121114327 (PMC3522916; doi:10.3390/s121114327)
Supplement: Supplementary file 1 [file sensors-12-14327-s001.pdf]

OPEN ACCESS

sensors

ISSN 1424-8220
www.mdpi.com/journal/sensors

Supplementary Information

Mass Transport Effects in Suspended Waveguide Biosensors Integrated in Microfluidic Channels. *Sensors* 2012, *12*, 14327-14343

Chaitanya R. Murthy and Andrea M. Armani *

Mork Family Department of Chemical Engineering and Materials Science,
University of Southern California, 3651 Watt Way, Los Angeles, CA 90089, USA;
E-Mail: cmurthy@usc.edu

* Author to whom correspondence should be addressed; E-Mail: armani@usc.edu;
Tel.: +1-213-740-4428; Fax: +1-213-740-7797.

1. Theory and Model

Our model involves three simultaneous and coupled processes: fluid flow, mass transport, and a first-order surface reaction. We must solve the time-dependent partial differential equations that govern each physical process. Even for the simplified 2-dimensional geometry described in the main paper, this would be a daunting problem. However, the scope of our study allows us to simplify the calculations required tremendously. Since we are dealing with bulk analyte concentrations in the sub-micromolar regime, the effect of mass transport on the fluid velocity is negligible, and we can split up the simulations into two stages: (1) solve for fluid flow and (2) use this result in solving the coupled transient mass transport and surface reaction equations. This approach leverages the ability of COMSOL Multiphysics to perform iterative simulations. In addition, we limit the range of flow velocities studied to fall within the laminar flow regime, which allows us to use a steady-state solution to Part 1.

1.1. Fluid Flow

The steady-state Navier-Stokes equations for an incompressible Newtonian fluid with no volume forces are:

$$\rho(v \cdot \nabla v) = \nabla \cdot [-p\mathrm{I} + \mu(\nabla v + (\nabla v)^T)] \quad \text{(S1)}$$

$$\nabla \cdot v = 0 \quad \text{(S2)}$$

where ρ is the fluid density, μ is the fluid viscosity, v is the fluid velocity vector, p is pressure, and I is the identity matrix. Equations (S1) and (S2) are expressions of conservation of fluid momentum and mass respectively.

In our model of a 2-dimensional cross-section of the flow cell, we neglect wall effects that might alter the velocity field in the width direction. This is an acceptable approximation when the effective sensing area of the waveguide is situated in the middle of the channel and away from the sidewalls, as it is for the devices of interest, or when the channel width is very large relative to the channel height. We solve Equations (S1) and (S2) simultaneously, subject to the following boundary conditions:

(BC-1) At all solid surfaces (*i.e.*, the top and bottom channel walls and the waveguide surface), we assume no-slip:

$$v = 0 \tag{S3}$$

(BC-2) At the channel inlet, we assume unidirectional pressure-driven flow with a parabolic velocity profile given by:

$$v(y) = \hat{x}\frac{\Delta P}{2\mu}y(H-y) \tag{S4}$$

where ΔP is the axial pressure gradient, H is the channel height, $\hat{x}$ is the unit vector in the direction of flow, and y is the coordinate in the direction of the cell height, with $y = 0$ at the cell floor. Integrating across the channel height, we obtain:

$$\overline{v}_{in}H = \int_0^H v(y)\cdot\hat{x}dy = \Delta P H^3/12\mu \tag{S5}$$

which can be inserted into (S4) to obtain the boundary condition equation used in our model, an inlet velocity field parameterized by $\overline{v}_{in}$:

$$v(y) = \hat{x}\frac{6\overline{v}_{in}}{H^2}y(H-y) \tag{S6}$$

(BC-3) At the channel outlet, we assume atmospheric pressure:

$$p = p_{atm} \tag{S7}$$

Solving Equations (S1) and (S2) using the boundary conditions described here gives the steady-state velocity profile and pressure distribution in the channel. These results are shown in Figure S1 for $\overline{v}_{in}$ = 0.4 m/s and suspended waveguide elevation of 28 μm.

Figure S1. Steady-state (**a**) velocity and (**b**) pressure profiles for flow around the suspended waveguide sensor, where average inlet flow velocity $\overline{v}_{in}$ = 0.4 m/s and waveguide elevation h = 28 μm as described in Figure 2(a) in the manuscript

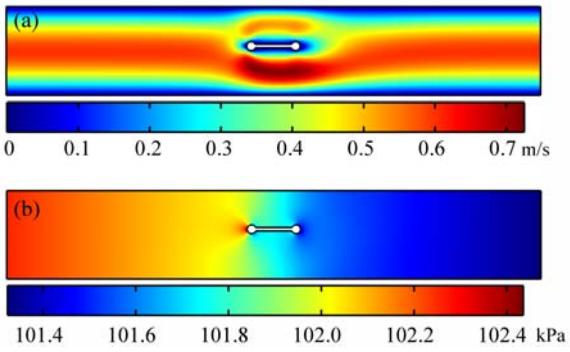

1.2. Mass Transport and Surface Reaction

The transport of analyte in bulk solution is described by the convection-diffusion equation:

$$\frac{\partial[\mathrm{A}]}{\partial t} + v \cdot \nabla[\mathrm{A}] = \nabla \cdot (D\nabla[\mathrm{A}]) \tag{S8}$$

where [A] is the analyte concentration, D is the diffusivity of the species, and $\boldsymbol{v}$ is the velocity field solved for numerically as in Section 1.1.

The binding of analyte to the functionalized sensor surface is assumed to follow a first-order Langmuir model describing the reaction between a freely-diffusing antigen A and an unoccupied binding site B, forming a bound complex C_s. This reaction may be expressed as:

$$\mathrm{A} + \mathrm{B} \rightleftharpoons \mathrm{C_s} \tag{S9}$$

and its forward ($rate_f$) and reverse ($rate_r$) reaction rates are given by:

$$rate_f = k_f[\mathrm{A}][\mathrm{B}] \tag{S10}$$

$$rate_r = k_r[\mathrm{C_s}] \tag{S11}$$

Here, k_f and k_r are the forward and reverse kinetic rate constants, respectively, and [ ] denotes species concentration. The concentration of unoccupied binding sites [B] is subject to conservation of species according to the expression:

$$[\mathrm{B}] = [\mathrm{B}]_m - [\mathrm{C_s}] \tag{S12}$$

where $[\mathrm{B}]_m$ is the total concentration of binding sites. Combining Equations (S10) through (S12), we obtain the following mass-balance expression for the surface concentration of bound antigen-antibody complex:

$$\frac{\partial[\mathrm{C_s}]}{\partial t} = k_f[\mathrm{A}]|_s([\mathrm{B}]_m - [\mathrm{C_s}]) - \mathrm{k}_r[\mathrm{C_s}] \tag{S13}$$

where $[\mathrm{A}]|_s$ is the concentration of analyte at the sensor surface. Equations (S8) and (S13) must be solved simultaneously to determine the time-dependent concentration fields. The following initial condition and boundary conditions apply:

(IC) At time $t = 0$, [A] = 0 and $[\mathrm{C_s}] = 0$ everywhere on the sensor surface.

For all $t > 0$,

(BC-1) At the channel inlet, we fix the analyte concentration at $[\mathrm{A}] = [\mathrm{A}]_0$

(BC-2) At the sensor surface, the following reaction/flux boundary condition couples Equations (S8) and (S13). The unit outward normal vector to the sensor surface, $\hat{n}$, is used to express the flux of analyte leaving solution as:

$$-\hat{n} \cdot ([\mathrm{A}]v - D\nabla[\mathrm{A}]) = \frac{\partial[\mathrm{C_s}]}{\partial t} \tag{S14}$$

(BC-3) The net mass flux across all other (non-binding) surfaces is zero:

$$-\hat{n} \cdot ([\mathrm{A}]v - D\nabla[\mathrm{A}]) = 0 \tag{S15}$$

(BC-4) There is no diffusive mass flux out of the flow cell (analyte is removed via advection only):

$$\hat{n} \cdot (-D\nabla[\mathrm{A}]) = 0 \tag{S16}$$

1.3. Laminar Flow Assumption

The Reynolds number:

$$\mathrm{Re} = \frac{v_0 l_0}{\nu} \tag{S17}$$

is a dimensionless number that arises naturally from the dimensionless form of Equation (S1). It gives an indication of the relative importance of inertial and viscous forces in a fluid system. Here, v_0 is a characteristic velocity, l_0 is a characteristic length scale, and $\nu = \mu/\rho$ is the kinematic viscosity (dynamic viscosity divided by density) of the fluid. In our case, we define separate Reynolds numbers for the channel and the sensor:

$$\mathrm{Re}_c = \frac{\overline{v}_{in} H}{\nu} \tag{S18}$$

and:

$$\mathrm{Re}_s = \frac{\overline{v}_{in} l}{\nu} \tag{S19}$$

where $\overline{v}_{in}$ is the mean inlet fluid velocity, H is the channel height, and $l = w + d$ is the length of the sensor in the direction of flow (see Figure 2(a) in the main text). Indicative values for the range of flowrates studied are listed in Table S1 below. For fully-developed flow in a channel, laminar flow occurs for $\mathrm{Re}_c < 2{,}000$, and we are well within this domain. We approximate our sensor as a cylinder in order to validate our assumption of laminar flow in the channel and use the literature analysis for flow around such an obstacle. At $\mathrm{Re}_s \ll 1$, flow is orderly, and at $\mathrm{Re}_s \sim 10$, a pair of stable vortices appears behind the sensor, but flow as a whole remains laminar [1]. The presence/absence of these vortices should not significantly affect mass transfer to the sensor, since they exist downstream of it.

Table S1. Reynolds numbers.

$\overline{v}_{in}$	Re_c	Re_s
0.5 m/s	25	15
5×10^{-3} m/s	0.25	0.15
5×10^{-5} m/s	2.5×10^{-3}	1.5×10^{-3}

2. Model Convergence

The finite element mesh used for this study was composed of triangular elements with sizes constrained by:

$$x_v^{max} = \frac{H}{n_v} \tag{S20}$$

and:

$$x_s^{max} = \frac{d}{2n_s} \tag{S21}$$

where x_v^{max} and x_s^{max} are the maximum allowed mesh element sizes in the flow cell and on the surface of the waveguide, respectively, H is the channel height, d is the diameter of the waveguide, and n_v and n_s are tunable mesh size parameters. By independently varying n_v and n_s, we control the quality of the mesh generated. A finer mesh produces a more precise solution, but can require vastly greater computation power and time to solve. The final mesh used in our study is shown in Figure S2 below. The mesh is characterized by $n_v = 40$ and $n_s = 40$, and contains 57,916 elements.

Figure S2. Representative finite element mesh used in this study. Naturally, when the sensor geometry was varied, the precise mesh generated by COMSOL under the given specifications changed slightly. (**a**) Image of entire flow cell (individual mesh elements may not be visible). (**b**) Close up of a region near the waveguide surface. (**c**) Further enlargement showing individual mesh elements on/near the waveguide surface.

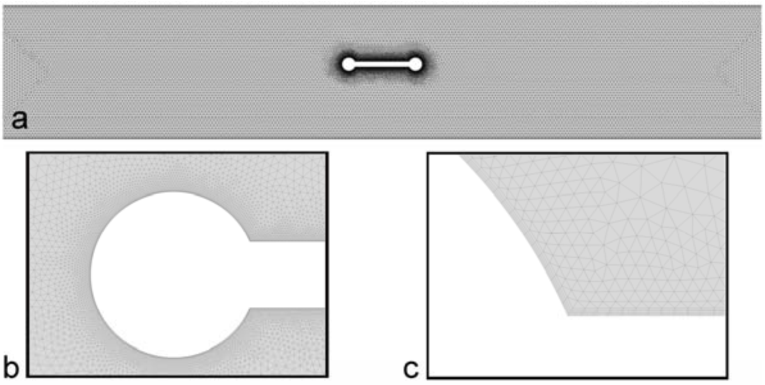

2.1. Determining Optimum n_v

The dominant processes occurring in the flow cell, far from the waveguide surface, are fluid flow and convective mass transport. Thus, a good criterion for evaluating the effectiveness of the mesh here is the resolution of the laminar flow profile generated. We evaluated the flow velocities along a cutline near the entrance to the flow cell (Figure S3(a)) while varying n_v, and compared these values to the smooth parabolic profile specified by the inlet boundary condition Equation (S6); n_s was held constant at 40. As Figure S3(b) shows, the profiles quickly approach the correct shape as n_v (and, consequently, the number of nodes along the cutline) increases. We quantify this convergence by calculating the root-mean-square error in each approximation of the flow profile (Figure S3(c)). Based on this data, we chose $n_v = 40$, which corresponds to ~0.1% RMS deviation from the predicted flow profile.

Figure S3. n_v convergence. (**a**) Schematic of flow cell showing cutline near inlet at which flow velocities were evaluated. (**b**) Flow profiles obtained along the cutline for various values of n_v ($\overline{v}_{in}$ = 0.5 m/s). The profiles clearly converge to a smooth parabola as n_v increases. (**c**) Root mean square deviation of the calculated flow field from the theoretical profile specified in Equation (S6) as a function of n_v at three $\overline{v}_{in}$ values spanning the range of flow velocities considered in our study.

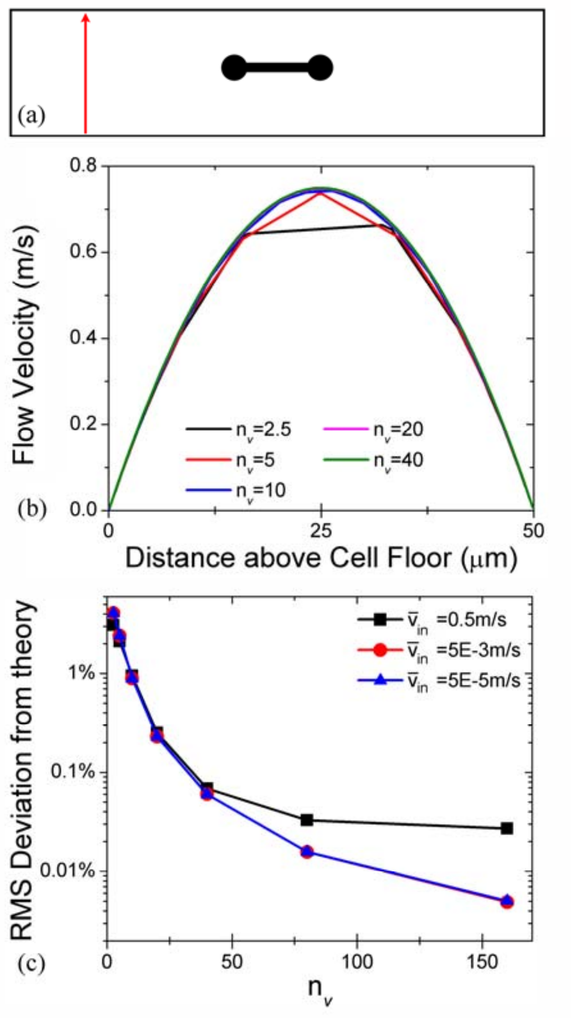

2.2. Determining Optimum n_s

We determined the effectiveness of the mesh at the waveguide surface by monitoring convergence to the known equilibrium concentration of bound analyte. At equilibrium, the amount of analyte bound per unit length of the sensor is given by:

$$\oint [\mathrm{C_s}]ds = \mathcal{P}_{wg}[\mathrm{C_s}]_{eq} \tag{S22}$$

where:

$$[\mathrm{C_s}]_{eq} = \frac{[\mathrm{A}]_0[\mathrm{B}]_m}{K_D + [\mathrm{A}]_0} \tag{S23}$$

and $\mathcal{P}_{wg}$ is the perimeter of the cross-section of the waveguide (~68.1356 μm). Figure S4(a) displays binding data obtained at different values of n_s (with n_v = 40, as determined in the preceding analysis). We can see that as we increase mesh resolution, the ratio of the (calculated) contour integral on the left to the (exact) expression on the right converges to 1. The percent deviation:

$$\left|1 - \frac{\oint [\mathrm{C_s}] ds}{\mathcal{P}_{wg}[\mathrm{C_s}]_{eq}}\right| \times 100 \tag{S24}$$

is plotted *versus* n_s in Figure S4(b). Based on this data, we chose n_s = 40, which corresponds to ~0.001% deviation from the accurate equilibrium concentration of bound analyte.

Figure S4. Verification of n_s convergence. (**a**) Binding data at a range of n_s values. Each curve asymptotically approaches an equilibrium amount of bound analyte as t increases. As n_s increases, these equilibrium amounts converge to the analytical value given by the right side of Equation (S22). (**b**) Percent deviation of the bound analyte concentration from the analytical equilibrium value as a function of n_s and time. Data is presented at different times to demonstrate that the degree of convergence can be arbitrarily improved by increasing n_s at large enough times.

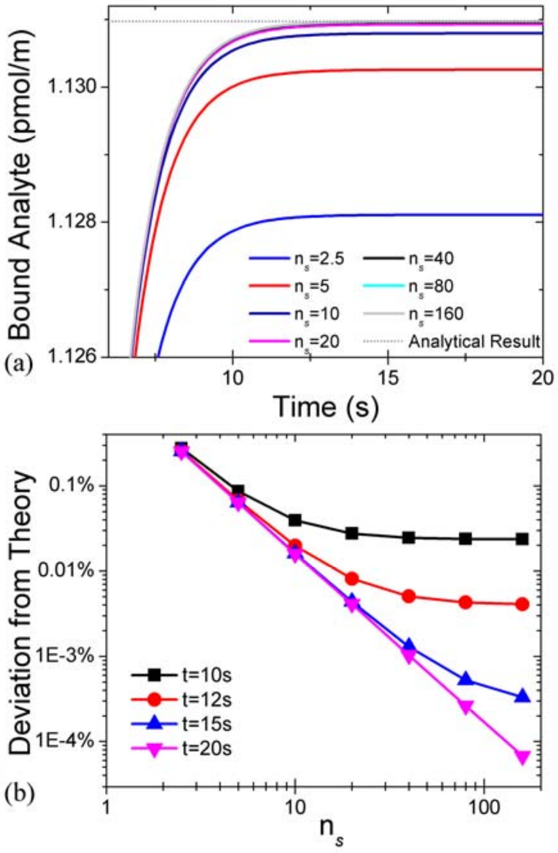

2.3. Convergence to $[C_s]_{eq}$

We quantify convergence to the theoretical equilibrium concentration by using the method described in Section 2.2. Specifically, we evaluate Equation (S24) at the last time step of each simulation performed. The results are displayed in Figure S5.

Figure S5. Percent deviation of the calculated average concentration of bound analyte at the last time step from the analytical value plotted as a function of average inlet flow velocity $\overline{v}_{in}$ for various parameter values. (**a**) Convergence to $[C_s]_{eq}$ for studies varying inlet bulk analyte concentration $[A]_0$ and total surface concentration of binding sites $[B]_m$ on the mid-channel suspended sensor. (**b**) Convergence to $[C_s]_{eq}$ for studies varying sensor elevation and geometry (inlet bulk analyte concentration $[A]_0$ = 100 nM and total surface concentration of binding sites $[B]_m$ = 1.66×10^{-8} mol/m^2). The sensor-surface mesh element size has been optimized to resolve binding to the complex suspended waveguide geometry. Therefore, the equivalent mesh is able to easily model binding to the simple planar geometry, resulting in very accurate solutions for these cases.

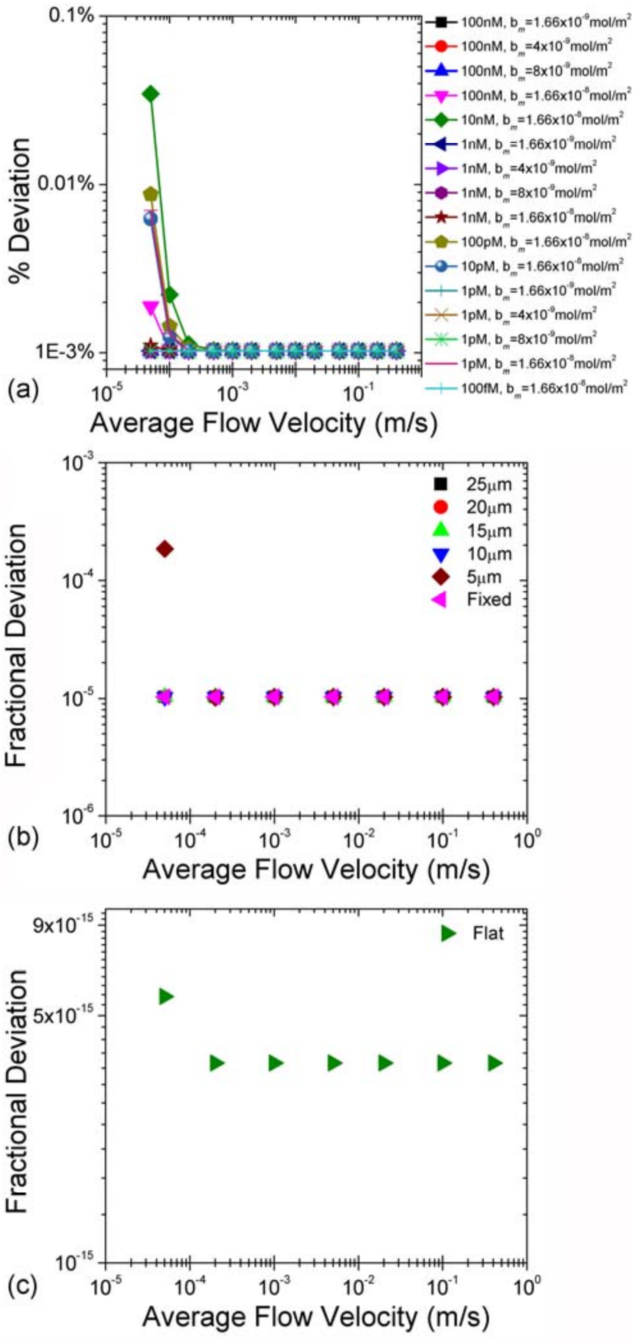

In most cases, the deviation is ~0.001%; the n_s-limited value determined in the convergence analysis of Section 2.2. The highest deviation is ~0.025%, and Figure S4(b) suggests that we could reduce this by simply extending those simulations to later times. Since our metric for equilibration is 95% of $[C_s]_{eq}$, however, it is unnecessary to do so, as the degree of asymptotic convergence demonstrated is more than sufficient (0.025% « 5%).

3. Additional Results

3.1. Start of Binding Time t_1

We define the start of binding as the time t_1 (and corresponding average surface concentration $[C_s]_1$) when, on average, a single analyte molecule is bound per micrometer length along the sensor. The number of bound analyte molecules per unit sensor length is given by:

$$N_C = N_A \oint [C_s] ds \tag{S25}$$

where N_A is Avogadro's constant, and the integral is over the perimeter of the waveguide cross-section. With $N_C = 10^6$ molecules/m, we get:

$$[C_s]_1 = \frac{10^6}{N_A \mathcal{P}_{wg}} \tag{S26}$$

Figure S6. Start-of-binding times t_1 as a function of average inlet flow velocity $\overline{v}_{in}$ and inlet bulk analyte concentration $[A]_0$.

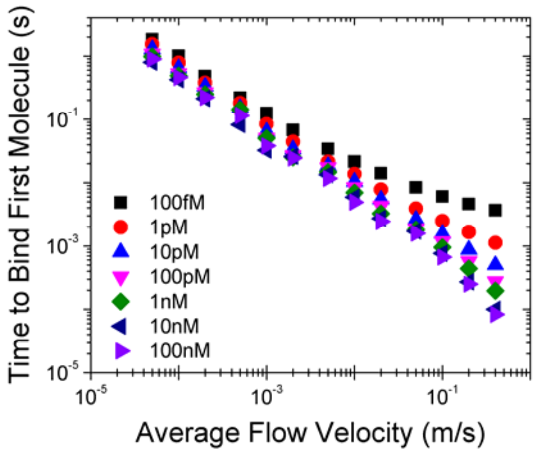

3.2. Effect of Detection Threshold on Sensor Elevation/Geometry Study Results

As discussed in the main text, we assumed an approximate detection threshold for the suspended waveguide sensor studied here based on previous reports involving similar devices. Figure S7 shows that the qualitative response behavior of the various sensor elevations and geometries remains consistent over a wide range of noise-limited detection thresholds. In all cases, the suspended sensors outperform the embedded ones, and performance increases as the waveguides are located nearer to mid-channel. The quantitative differences in detection times are greatest for low flow velocities and high detection limits.

Figure S7. Relative detection times as a function of sensor elevation/geometry and Sensor Péclet Number $\text{Pe}_l = \overline{v}_{in} l/D$ for various detection thresholds (inlet bulk analyte concentration $[\text{A}]_0$ = 100 nM and total surface concentration of binding sites $[\text{B}]_m = 1.66 \times 10^{-8}$ mol/m^2 in all cases). Consult Figure 2 in the main text for schematics of the geometries considered. The detection thresholds considered are integer multiples or fractions of the value defined in the manuscript: $N_d = 1.58 \times 10^7$ molecules/mm. (**a**) $0.2N_d$; (**b**) $0.5N_d$; (**c**) $2N_d$; (**d**) $5N_d$. Compare with Figure 7(a) in the main text, where the detection threshold is $1N_d$.

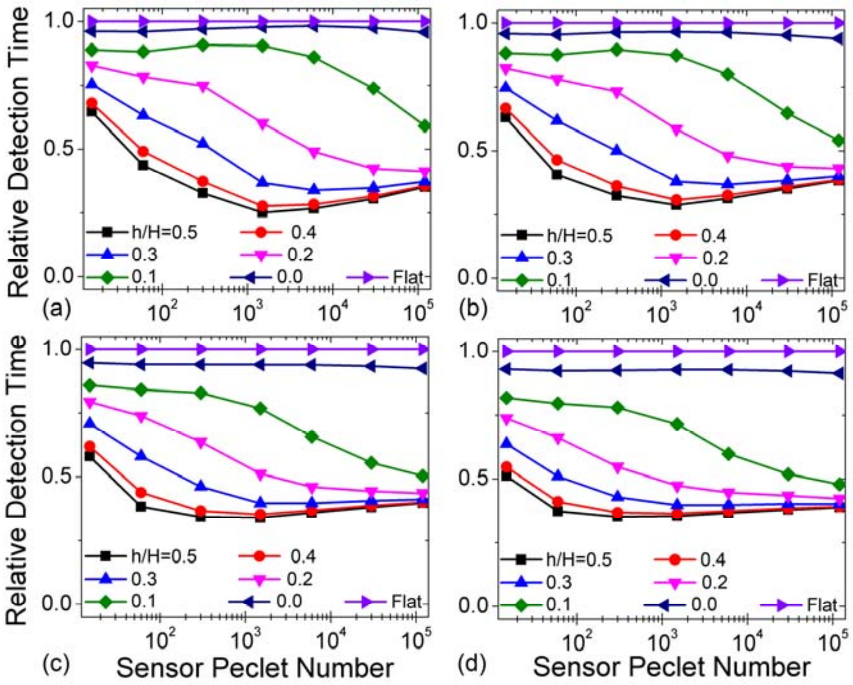

4. Derivations

4.1. Slow-Limit Velocity Expression

The average velocity of pressure-driven flow through a channel of height h is given by:

$$\overline{v}_h = \Delta P h^2 / 12\mu \tag{S27}$$

For our hypothetical two-channel system, neglecting the thickness of the sensor itself, we have:

$$H = h_1 + h_2 \tag{S28}$$

A mass balance over the whole system gives:

$$\overline{v} H = \overline{v}_1 h_1 + \overline{v}_2 h_2 \tag{S29}$$

In the slow limit, ΔP is identical in both hypothetical channels (as is μ, of course). Substituting Equation (S27) into (S29):

$$\overline{v} H = \frac{\Delta P}{12\mu}\left({h_1}^3 + {h_2}^3\right) \tag{S30}$$

Therefore:

$$\frac{\overline{v}_1}{\overline{v}} = \frac{{h_1}^2 H}{\left({h_1}^3 + {h_2}^3\right)} \tag{S31}$$

which can be rearranged to give Equation (5) using the identity Equation (S28).

4.2. Fast-Limit Velocity Expression

In the fast limit, the total flowrate through the region above or below the sensor is found simply by integrating the inlet parabolic velocity profile Equation (S6) over that part of the channel:

$$\overline{v}_1 h_1 = \int_0^{h_1} \frac{6\overline{v}_{in}}{H^2} y(H - y)\, dy \tag{S32}$$

Evaluating the integral and rearranging gives Equation (6) directly.

Reference

1. Bird, R.B.; Stewart, W.E.; Lightfoot, E.N. *Transport Phenomena*, 2nd revised ed.; John Wiley & Sons: New York, NY, USA, 2007.

© 2012 by the authors; licensee MDPI, Basel, Switzerland. This article is an open access article distributed under the terms and conditions of the Creative Commons Attribution license (http://creativecommons.org/licenses/by/3.0/).
